# Factors associated with diabetic retinopathy among patients with diabetes in rural Guangxi, China: a multicenter cross-sectional study

**DOI:** 10.3389/fendo.2026.1683063

**Published:** 2026-01-30

**Authors:** Xiaomin Xian, Jingfeng Chen, Ziqiang Li, Qiuping Zheng, Xiaoxue Lei, Chaoqun Bai, Yanping Zhang, Guifen Fu

**Affiliations:** 1 Nursing Department, Faculty of Chinese Medicine Science, Guangxi University of Chinese Medicine, Nanning, China; 2 Department of Geriatric Endocrinology and Metabolism, Guangxi Academy of Medical Sciences and the People’s Hospital of Guangxi Zhuang Autonomous Region, Nanning, China; 3 Department of Nursing, Guangxi Academy of Medical Sciences and the People’s Hospital of Guangxi Zhuang Autonomous Region, Nanning, China

**Keywords:** correlates of DR, diabetes, prevalence, retinopathy, rural

## Abstract

**Objective:**

The primary objective of this study was to estimate the prevalence of diabetic retinopathy (DR) among adult patients with diabetes in rural Guangxi. The secondary objective was to identify demographic, clinical, and psychosocial factors associated with DR. These findings could offer practical evidence for improving diabetes management in remote areas.

**Methods:**

From January to July 2022, this population-based multicenter cross-sectional study employed a multi-stage stratified sampling method. Initially, five cities were randomly selected within the region. Next, three counties were randomly chosen from each city, totaling 15 counties. Data on sociodemographic characteristics, diabetes-related factors, and laboratory indicators were collected using questionnaires and clinical examinations. DR was assessed through dilated fundus examination or fundus photography. Multivariable logistic regression was performed to estimate adjusted odds ratios (ORs) and identify factors associated with DR.

**Results:**

A total of 2,178 patients with diabetes were included, comprising 1,204 males (55.28%) and 974 females (44.72%), with a mean age of 63.25 ± 12.71 years. The prevalence of DR among people living with diabetes in rural Guangxi was 22.36% (95% *CI*: 20.62%–24.10%). Multivariate logistic regression analysis revealed that advanced age (*OR* = 1.015, 95%*CI:* 1.001–1.029, *P* = 0.042), increased HbA1c levels (*OR* = 4.004, 95% *CI*: 3.435–4.666, *P* < 0.001), and elevated triglyceride levels (*OR* = 1.493, 95% *CI*: 1.267–1.758, *P* < 0.001) were significantly associated with the development of DR in patients with diabetes.

**Conclusion:**

The prevalence of DR among people living with diabetes in rural Guangxi was 22.36%. This finding underscores the importance of healthcare providers closely monitoring older adults and patients with elevated HbA1c and triglyceride levels.

## 1 Introduction

Diabetes mellitus is a metabolic disorder characterized by chronic hyperglycemia resulting from impaired insulin secretion or action (1). With aging populations and increased life expectancy, diabetes has become a major global public health challenge (2). According to the IDF Diabetes 2021, approximately 537 million adults worldwide had diabetes, with a projected increase to 783 million by 2045 (3). Persistent hyperglycemia can lead to macrovascular and microvascular complications. Diabetic retinopathy is the most common microvascular complication and a leading cause of visual impairment and blindness (4, 5). Previous studies show that the prevalence of DR varies significantly across regions and socioeconomic contexts. For instance, a hospital-based study in Bhutan reported DR prevalence reaching 42.7% among people living with diabetes, with diabetic macular edema present in 14% of cases (6). In contrast, research conducted in Spain, a developed nation, reported a prevalence of 12.3% (95% *CI*: 12.1%–12.5%) for any DR type (7). These differences suggest that variations in healthcare access, screening programs, and glycemic control across countries and regions significantly influence DR epidemiology. Globally, the prevalence of DR among patients with diabetes is approximately 34.6%, with vision-threatening DR accounting for 10.2% (8). In China, the prevalence is about 16.3% (95% *CI*: 15.3%–17.2%) (9). DR is often asymptomatic initially but can lead to severe visual impairment and increase the risk of other complications as the disease advances (10, 11).

Rural Guangxi, an economically disadvantaged area, faces substantial diabetes and DR burdens due to residents’ lower education levels and limited healthcare access (12). Barriers such as inadequate transportation, financial hardships, and lack of routine eye exams frequently delay DR diagnosis, causing irreversible visual loss (13). Additionally, poor diabetes awareness and glycemic control among rural people living with diabetes further complicate DR prevention and management (14–16). Although earlier studies indicate relationships between DR and factors such as hyperglycemia, hypertension, higher cholesterol levels, diabetes duration, and obesity (17, 18), most of this evidence originates from Western countries. Several studies in China have investigated the prevalence and correlates of DR, but these have mainly concentrated on populations from northern and southern China (19, 20). Considering China’s considerable diversity in dietary habits, healthcare resources, socioeconomic conditions, and diabetes management approaches, unique regional factors associated with DR likely exist. However, robust multicenter studies simultaneously evaluating clinical indicators, diabetes-related knowledge, and social support factors related to DR remain limited.

Therefore, this multicenter cross-sectional study aimed to estimate DR prevalence and explore its associated factors, thereby providing evidence to inform targeted prevention strategies and enhance early diagnosis and management.

## 2 Materials and methods

### 2.1 Participants

**Figure 1:**
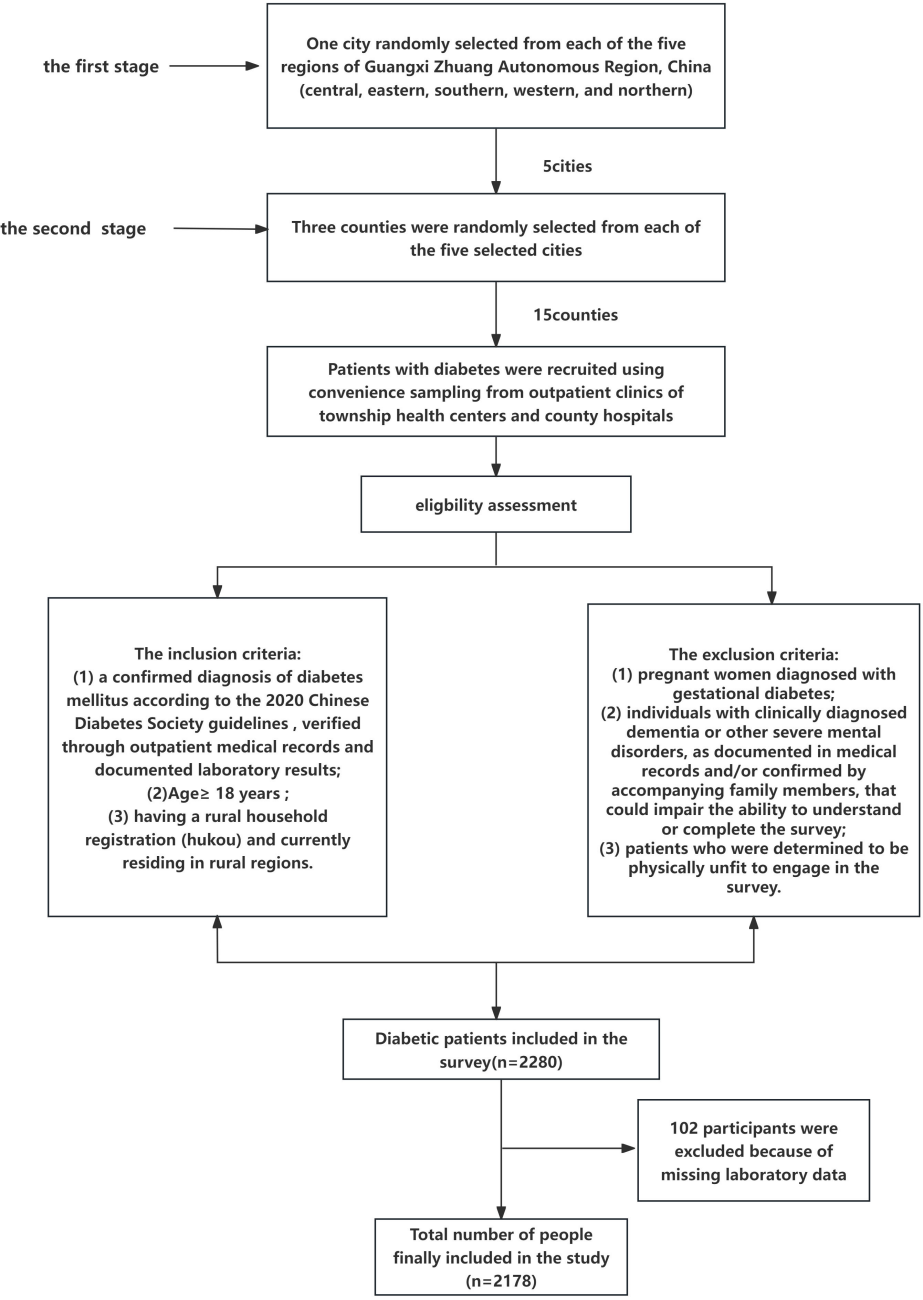
Flowchart of a sample of a people living with diabetes.

This multicenter cross-sectional study was conducted from January to July 2022 to assess diabetes-related complications among patients with diabetes in rural areas of the Guangxi Zhuang Autonomous Region, China. A multistage stratified sampling design was used to select representative samples. In the first stage, one city was randomly selected from each geographic region (central, eastern, southern, western, and northern). In the second stage, three counties were randomly selected from each city, resulting in 15 counties included in the survey. In each selected county, a facility-based convenience sampling strategy was adopted to recruit patients with diabetes, including both outpatients and inpatients, attending township health centers and county hospitals. Eligible participants were identified through the diabetes management system of the National Basic Public Health Service Program, which maintains a registry of diagnosed patients with diabetes. Therefore, while cities and counties were randomly selected, participant recruitment within facilities was non-probabilistic, and the sample primarily represents health-seeking, registered rural patients with diabetes rather than the entire rural diabetic population. The inclusion criteria were as follows (1): a confirmed diagnosis of diabetes mellitus according to the 2020 Chinese Diabetes Society guidelines (21), verified by outpatient medical records and laboratory findings (2); age ≥18 years; (3) rural household registration (hukou) with current residence in rural areas. The exclusion criteria were as follows: (1) pregnant women with gestational diabetes; (2) individuals with clinically diagnosed dementia or other severe mental disorders, documented in medical records and/or confirmed by family members, that could limit comprehension or survey completion; (3) patients deemed physically unable to participate. The participant selection process is illustrated in **Figure 1**.

### 2.2 Sample size

In a preliminary survey, 150 rural patients with diabetes were enrolled, with 10 participants recruited from each of the 15 counties. Among these individuals, 116 had diabetes-related complications, yielding a prevalence of 77.33%. This value was used as the anticipated prevalence (P) for sample size estimation. The sample size was calculated using the standard formula for multistage stratified sampling (22): n=*Z*²_1-α/2_(*1-P*)*P*/*δ*² × *deff* (where *Z*_1-α/2_ = 1.96; *P* is the prevalence, which was 77.33% in this study; *deff* represents the design effect, which was set to 2 to account for regional variability, and*δ* denotes the allowable error, set at 0.1. Because this was a county-level multistage stratified survey in rural settings, the expected prevalence was defined as the prevalence of overall diabetes-related complications, for which reliable pilot data were available. Based on these data, *P* was fixed at 77.33%. The tolerable relative error (*δ*) corresponded to a precision of 10%, a value commonly applied in large-scale epidemiological surveys. A design effect of 2 was incorporated to account for clustering and inter-county heterogeneity. Under these assumptions, the minimum required sample size was estimated at 136 patients with diabetes per county. To compensate for potential non-response and incomplete data, the sample size was increased by 10%, yielding a final target of 152 participants per county. Consequently, 2,280 patients with diabetes were targeted across the 15 counties. Although the calculation was based on the prevalence of overall diabetes-related complications, the achieved sample size was sufficient to ensure robust estimation of DR prevalence and adequate statistical power for subsequent multivariable analyses.

### 2.3 Research tools

#### 2.3.1 General social information survey

Socio-demographic information included individual characteristics such as gender, education level, marital status, occupation, annual per capita disposable household income (23), and drinking behavior. Disease-related information included glycated hemoglobin (HbA1c) levels, use of hypoglycemic drugs, insulin administration, complications, and family history of diabetes mellitus. In this study, the definitions of insulin and diabetes drug use were as follows (24): Insulin users were defined as patients currently undergoing insulin treatment, regardless of whether they were also taking oral antidiabetic medications; diabetes drug users were defined as patients who took only oral antidiabetic medications without insulin.

#### 2.3.2 Diabetes knowledge assessment score

The Diabetes Knowledge Questionnaire, developed by Speight and Bradley at the University of London in 2001 (25), covers diabetes-related knowledge, including treatment methods, dietary management, exercise, hypoglycemia management, foot care, consequences of smoking and drinking, illness management, and complication risk reduction. The questionnaire comprises 26 subdimensions and 110 items, each offering three response options: “correct,” “incorrect,” and “don’t know.” Each correct response is scored 1 point, while incorrect or “don’t know” responses receive 0 points. The total score, calculated by summing all items across the subdimensions, indicates the respondent’s diabetes knowledge level, with higher scores reflecting greater knowledge. In 2003, Weiyan et al. (26) assessed the reliability and validity of the Chinese version of the Diabetes Knowledge Scale. The Cronbach’s α coefficient of this scale was 0.909, indicating excellent reliability, validity, stability, and strong internal consistency.

#### 2.3.3 Social support index

The Social Support Rating Scale (SSRS), developed by Shuiyuan et al. (27), assesses three dimensions: subjective support, objective support, and utilization of support. Total scores obtained by summing responses across these subdimensions indicate overall levels of social support, with higher scores signifying greater perceived support. This instrument demonstrated strong reliability, as indicated by a Cronbach’s α coefficient of 0.838.

#### 2.3.4 Assessment of DR

In accordance with diagnostic criteria and screening methods recommended by the Chinese Guidelines for the Prevention and Treatment of Type 2 Diabetes (21), collaborative screening for diabetic complications was conducted by medical personnel from 15 participating hospitals. Specifically, trained ophthalmologists assessed DR. The screening covered DR, nephropathy, neuropathy, lower extremity arterial disease, and diabetic foot. DR diagnoses were confirmed by reviewing relevant patient medical records, typically documented during routine ophthalmologic examinations at county or township hospitals. DR was identified through dilated fundus examination and/or fundus photography.

In this study, DR data collection took place during routine outpatient visits, utilizing dilated fundus examination and/or fundus photography. Due to practical limitations inherent in outpatient screening, comprehensive severity grading of DR and evaluation of inter-observer reliability were not feasible. Therefore, DR status was defined dichotomously (“any DR”) for statistical analysis, indicating only presence or absence.

#### 2.3.5 Laboratory assessments

On the day following admission, medical staff at each participating hospital collected 2 mL of venous blood from patients after a 10-hour fasting period. All biochemical analyses were conducted in clinical laboratories following standardized protocols. The blood samples were centrifuged at 3,000 × g for 10 minutes to separate the plasma. Nurses and laboratory technicians assessed the following indicators: HbA1c levels were measured by high-performance liquid chromatography using Bio-Rad Variant II analyzers (Bio-Rad Laboratories, USA) according to the manufacturer’s instructions. Lipid profiles, including total cholesterol (TC), triglycerides (TG), high-density lipoprotein cholesterol (HDL-C), and low-density lipoprotein cholesterol (LDL-C), were determined enzymatically using Mindray BS-800 automatic biochemical analyzers (Mindray Medical International, Shenzhen, China) with manufacturer-matched reagents (TC: MEI-00423; TG: MEI-00424; HDL-C: MEI-00426; LDL-C: MEI-00427). Serum creatinine and uric acid concentrations were analyzed spectrophotometrically using commercial assay kits (Leadman Biochemistry, Beijing, China). According to the Chinese treatment guidelines for type 2 diabetes (21), the recommended blood lipid targets for patients with diabetes are as follows: TC <4.5 mmol/L; HDL-C >1.0 mmol/L (men) and HDL-C >1.3 mmol/L (women); TG <1.7 mmol/L; and LDL-C <1.8 mmol/L.

### 2.4 Variables definition

Smoking status was determined based on cumulative tobacco consumption lasting six months or longer. Participants were categorized into regular smokers (≥1 cigarette per day), occasional smokers (less than one cigarette per day, approximately weekly but not daily), and former smokers (cessation for >6 months). Alcohol intake was similarly classified as regular drinking (multiple times weekly but not daily), occasional drinking (weekly or less), and former drinking (abstinence for >6 months). For statistical analyses, smoking and alcohol consumption behaviors were simplified into binary categories: “yes” (current regular users) and “no” (never, occasional, or former users).

### 2.5 Data acquisition

Data collection methods in this study included a questionnaire survey and blood analysis. Primary healthcare personnel assisted patients in completing standardized questionnaires. They documented HbA1c, TC, TG, HDL-C, LDL-C levels, and complication screening results for people living with diabetes from 15 hospitals. Subsequently, these data were entered into Excel spreadsheets and transmitted electronically to the research team. The research team integrated data from all hospitals into a single Excel spreadsheet. Two researchers independently verified all data before entry into SPSS software for statistical analysis. All data are securely stored on confidential computers to prevent data leakage.

### 2.6 Validity, reliability, and rigor

This study employed a stringent quality control system. Firstly, all researchers received standardized training to ensure a thorough understanding of the research objectives and uniformity in data collection procedures. The research team carefully selected responsible and conscientious personnel to supervise data collection and management processes. Additionally, the team collaborated closely with the 15 participating hospitals, providing hospital managers with detailed explanations of the study objectives, diabetic complication screening protocols, questionnaire content, and instructions. This ensured the consistency and accuracy of data collected at each site. After data collection, the research team carefully evaluated and organized the data, working with field investigators to verify any abnormal entries. These procedures ensured the validity and reliability of all datasets.

### 2.7 Statistical analysis

This study utilized Microsoft Excel to construct a database, and statistical analyses were performed using SPSS version 25.0. Continuous variables, including age, HbA1c, creatinine, and total scores from the Diabetes Knowledge Questionnaire and the Social Support Rating Scale, were expressed as the mean ± standard deviation (SD) when normally distributed, and between-group comparisons were conducted using independent-samples t-tests. Non-normally distributed continuous variables were described using the median (interquartile range). Categorical variables were summarized as frequencies and percentages, and group comparisons were conducted using the chi-square test. Variables showing statistical significance in univariate analyses were subsequently included in binary logistic regression models. Variables exhibiting statistical significance (*P* < 0.05) in univariate analysis were subsequently incorporated into binary logistic regression models (**Table 1** lists these variables). To ensure model stability and prevent overfitting, only variables significant (*P* < 0.05) in univariate analyses were included in the multivariable logistic regression analysis. This methodological strategy balanced model simplicity and statistical robustness.

**Table 1:** Assigning values to variables.

Variables	Assigning values
Gender	Female = 0; Male = 1
Educational level	Illiterate = 0; Elementary school degree = 1; Junior high school degree = 2; Senior high school degree = 3; Associate degree = 4; Bachelor degree and above = 5
Household disposable income per capita	<18,931 CNY (2631USD) = 0; ≥18,931 CNY (2631USD) = 1
Marital status	Unmarried or widowed = 0; Married = 1
Type of diabetes	Special type of diabetes = 0; Type 1 diabetes = 1; Type 2 diabetes = 2
Duration of diabetes	≤5 years = 0; 5–10 years = 1;≥10 years =2
Family history of diabetes	No = 0; Yes = 1
Use diabetes drug	No = 0; Yes = 1
Use insulin	No = 0; Yes = 1
Smoking	No = 0; Yes = 1
Drinking	No = 0; Yes = 1
Diabetic nephropathy	No = 0; Yes = 1
Diabetic retinopathy	No = 0; Yes = 1
Diabetic peripheral neuropathy	No = 0; Yes = 1
Diabetic lower limb arteriopathy	No = 0; Yes = 1
Diabetic foot	No = 0; Yes = 1

### 2.8 Ethical considerations

Ethical approval was obtained from the Ethics Committee of Guangxi Zhuang Autonomous Region People’s Hospital (Approval number: KY-KJT-2021-26). Participation in the study was explicitly voluntary, as indicated clearly in the study questionnaires. Confidentiality and anonymity of patient data were strictly maintained. Written informed consent was obtained from all study participants as well as from hospital management representatives. No financial incentives were offered to participants.

## 3 Results

### 3.1 Basic information of patients

From the initial 2,280 rural people living with diabetes surveyed, 2,178 individuals were included in the final analysis, with 102 participants excluded due to incomplete laboratory data. The average age of the included participants was 63.25 ± 12.71 years. Male participants accounted for 55.28%, and married individuals comprised 92.75% of the sample. Regarding lifestyle habits, 18.19% (*n* = 396) reported a smoking history, and 22.77% reported alcohol consumption. With respect to diabetes management, 61.94% used oral hypoglycemic medications, while insulin therapy was used by 41.92% of participants. Additionally, 26.58% (*n* = 579) had a diabetes duration exceeding 10 years, and those with education limited to junior high school accounted for 31.40%. An annual household income equal to or greater than 18,931 CNY was reported by 37.74% (n=822), and a familial history of diabetes was noted in 14.55% of respondents. A substantial majority (77.32%, n=1,684) had suboptimal glycemic control. The diabetes type distribution was as follows: type 1 diabetes (1.97%), type 2 diabetes (97.47%), and special type of diabetes (0.56%). The overall complication prevalence reached 72.13% (*n* = 1,571), comprising DR (22.36%, *n* = 487), diabetic nephropathy (23.32%, *n* = 508), diabetic foot (9.83%), and lower extremity arterial disease (24.56%, *n* = 535). Because multiple complications could occur simultaneously in individual patients, the reported complication categories were not mutually exclusive. Detailed participant demographics are summarized in **Table 2**.

**Table 2:** General information characteristics of rural diabetic patients.

Variables	categorization	*N*(%)	*Mean (SD)*
Age			63.25±12.71
Gender	Male	1,204(55.28)	
	Female	974(44.72)	
Marital status	Married	2,020(92.75)	
	Unmarried or widowed	158(7.25)	
Educational level	Illiterate	256(11.75)	
	Elementary school degree	504(23.14)	
	Junior high school degree	684(31.41)	
	Senior high school degree	507(23.28)	
	Associate degree	180(8.26)	
	Bachelor degree and above	47(2.16)	
Smoking			
	No	1,782(81.82)	
	Yes	396(18.18)	
Drinking			
	No	1,682(77.23)	
	Yes	496(22.77)	
*Per capita* annual disposable income	<18,931 CNY (2631USD)	1,356(62.26)	
	≥18,931 CNY (2631USD)	822(37.74)	
Family history of diabetes			
	No	1,861(85.45)	
	Yes	317(14.55)	
Duration of diabetes			
	≤5 years	465(21.35)	
	5-10 years	1,134(52.07)	
	≥10years	579(26.58)	
Use diabetes drug			
	No	829(38.06)	
	Yes	1,439(61.94)	
Use insulin			
	No	1,265(58.08)	
	Yes	913(41.92)	
Glycosylated hemoglobin(%)	<7.0	494(22.68)	
	≥7.0	1,684(77.32)	
Type of diabetes			
	Type 1 diabetes	43(1.97)	
	Type 2 diabetes	2,123(97.47)	
	Special type of diabetes	12(0.56)	
Complications of diabetes			
	Diabetic nephropathy	508(23.32)	
	Diabetic retinopathy	487(22.36)	
	Diabetic peripheral neuropathy	635(29.15)	
	Diabetic lower limb arteriopathy	535(24.56)	
	Diabetic foot	214(9.83)	

### 3.2 Comparative analysis of patients with and without DR

**Table 3** summarizes demographic and biochemical characteristics of 487 DR patients compared with 1,691 people living with diabetes without retinopathy. Patients with DR had significantly older ages (66.17 ± 12.424 years) compared to those without DR (62.41 ± 12.673 years; *P* < 0.001). In addition, the univariate analyses demonstrated significantly higher prevalence of DR among male participants (*P* = 0.010), individuals with lower education (*P* < 0.001), those with annual per capita household disposable incomes below 18,931 CNY (*P* < 0.001), smokers (*P* = 0.014), longer diabetes durations (*P* < 0.001), patients using oral antidiabetic medications (*P* < 0.001) or insulin (*P* < 0.001), elevated HbA1c (*P* < 0.001), lower diabetes knowledge scores (*P* < 0.001), reduced social support (*P* < 0.001), higher creatinine levels (*P* < 0.001), increased low-density lipoprotein cholesterol (LDL-C, *P* < 0.001), and higher triglyceride levels (*P* < 0.001). However, marital status, alcohol consumption, familial diabetes history, uric acid, total cholesterol, and high-density lipoprotein cholesterol (HDL-C) showed no significant associations with DR in univariate analysis. Variables identified as statistically significant in univariate analyses were included in the multivariable logistic regression analysis. In the adjusted model, only age, HbA1c, and triglyceride levels remained significant independent correlates of DR (all *P* < 0.05). Detailed outcomes are presented in **Table 4**.

**Table 3:** Baseline characteristics of participants with and without diabetic retinopathy in rural areas.

Variables	N	Detinopathy (N=487) Mean ± SD	Non-retinopathy (N=1,691) Mean ± SD	Statistics	*P-*value
Age(year)		66.170 ± 12.424	62.410 ± 12.673	5.787^a^	<0.001
Gender				6.572^b^	0.010
Male	1,204	294	910		
Female	974	193	781		
Educational level				79.434^b^	<0.001
Illiterate	256	101	155		
Elementary school degree	504	136	368		
Junior high school degree	684	144	540		
Senior high school degree	507	81	426		
Associate degree	180	22	158		
Bachelor degree and above	47	3	44		
Marital status				2.727^b^	0.099
Married	2,020	460	1,560		
Unmarried or widowed	158	27	131		
Household disposable income per capita				41.606^b^	<0.001
<18,931 CNY (2631USD)	1,356	364	992		
≥18,931 CNY (2631USD)	822	123	699		
Smoking				6.055^b^	0.014
No	1,782	380	1,402		
Yes	396	107	289		
Drinking				3.426^b^	0.640
No	1,682	361	1,321		
Yes	396	126	370		
Family history of diabetes				1.078^b^	0.299
No	1,861	409	1,452		
Yes	317	78	239		
Duration of diabetes(year)				35.948^b^	<0.001
0-5	465	71	394		
5-10	1,134	240	894		
≥10	579	176	403		
Use diabetes drug				55.974^b^	<0.001
No	829	256	573		
Yes	1,412	231	1,118		
Use insulin				84.401^b^	<0.001
No	1,265	371	894		
Yes	913	116	797		
Glycosylated hemoglobin(%)		11.208 ± 1.518	7.854 ± 1.721	38.859^b^	<0.001
Creatinine		118.312 ± 63.030	105.453 ± 59.139	4.166^a^	<0.001
Uric acid		312.181 ± 108.991	302.872 ± 110.914	1.638^a^	0.102
Score of diabetes knowledge		43.80 ± 4.385	47.88 ± 4.405	18.007^a^	<0.001
Score of Social support score		21.92 ± 6.507	28.08 ± 10.313	12.475^a^	<0.001
total cholesterol		5.401 ± 2.557	5.226 ± 2.337	1.422^a^	0.155
triglycerides		2.446 ± 0.861	2.186 ± 1.003	5.152^a^	<0.001
High density lipoprotein		1.202 ± 0.358	1.169 ± 0.349	1.861^a^	0.063
low density lipoprotein		2.963 ± 0.951	2.843 ± 1.062	2.242^a^	0.025

a: t-test; b: chi-square test

### 3.3 Logistic regression study on factors associated with DR among rural patients with diabetes

In this study, DR was defined as the dependent variable (0 = no, 1 = yes). Age, sex, disease duration, smoking status, education level, annual per capita disposable household income, use of oral hypoglycemic agents, insulin therapy, HbA1c level, total diabetes knowledge score, total social support score, creatinine level, triglyceride level, and LDL-C level were included as independent variables (**Table 4**). Multivariable logistic regression analysis identified advanced age, elevated HbA1c levels, and increased triglyceride levels as the primary factors associated with DR among individuals with diabetes. Each additional year of age was associated with a 1.5% increase in the risk of DR (*OR* = 1.015, 95% *CI* = 1.001–1.029, *P* = 0.042). Individuals with higher HbA1c levels exhibited an approximately fourfold increased risk of DR compared with those with lower HbA1c levels (*OR* = 4.004, 95%*CI=*3.435–4.666, *P* < 0.001). Furthermore, each one-unit increase in triglyceride level was associated with a 49.3% increase in DR risk (*OR* = 1.493, 95%*CI=*1.267–1.758, *P* < 0.001).

**Table 4:** Logistic regression analysis of factors influencing DR in rural diabetic patients.

Variables	β	SE	*Wald χ2*	*P*	OR(95%CI)
Constants	-13.216	1.711	59.675	<0.001	–
Age(year)	0.015	0.007	4.123	0.042	1.015(1.001,1.029)
Gender					
Female(reference)	–	–	–	–	–
Male	-0.009	0.166	0.003	0.956	0.991(0.716,1.372)
Educational level(Overall Wald test)			5.366	0.373	
Illiterate(Ref.)	–	–	–	–	–
Elementary school degree	-0.663	0.795	0.696	0.404	0.515(0.109,2.447)
Junior high school degree	-0.931	0.782	1.416	0.234	0.394(0.085,1.826)
Senior high school degree	-1.118	0.779	2.058	0.151	0.327(0.071,1.506)
Associate degree	-0.842	0.780	1.166	0.280	0.431(0.093,1.987)
Bachelor degree and above	-1.022	0.827	1.529	0.216	0.360(0.071,1.818)
Household disposable income per capita					
<18,931 CNY (2631USD)(Ref.)	–	–	–	–	–
≥18,931 CNY (2631USD)	-0.122	0.177	0.479	0.489	0.885(0.625,1.252)
Smoking					
No(Ref.)	–	–	–	–	–
Yes	-0.357	0.198	3.259	0.071	0.700(0.475,1.031)
Duration of diabetes(year, (Overall Wald test)			0.167	0.920	
0-5(Ref.)	–	–	–	–	–
5-10	0.051	0.239	0.045	0.833	1.052(0.659,1.680)
≥10	-0.036	0.179	0.040	0.842	0.965(0.679,1.372)
Use diabetes drug					
No(Ref.)	–	–	–	--	–
Yes	-0.148	0.159	0.872	0.350	0.862(0.632,1.177)
Use insulin	-0.236	0.177	1.787	0.181	0.790(0.559,1.116)
No(Ref.)	–	–	–	–	–
Yes	-0.236	0.177	1.787	0.181	0.790(0.559,1.116)
Glycosylated hemoglobin(%)	1.387	0.078	315.236	<0.001	4.004(3.435,4.666)
Creatinine	-0.002	0.001	3.397	0.065	0.998(0.995,1.000)
Score of diabetes knowledge	-0.038	0.025	2.254	0.133	0.963(0.916,1.012)
Score of social support score	-0.006	0.012	0.274	0.601	0.994(0.970,1018)
Triglycerides	0.400	0.084	22.980	<0.001	1.493(1.267,1.758)
Low density lipoprotein	0.122	0.075	2.635	0.105	1.129(0.975,1.308)

*OR*, odds ratio; *CI*, confidence interval. Reference categories are indicated as “Ref”. *Overall Wald χ²* tests are presented for categorical variables where applicable.

## 4 Discussion

DR represents one of the most prevalent and severe ocular complications among individuals with diabetes. As the disease progresses to the proliferative stage, patients may experience visual impairment, visual field defects, and vitreous hemorrhage. Without timely intervention, DR may lead to tractional retinal detachment and neovascular glaucoma, ultimately resulting in blindness. DR has become the leading cause of vision impairment among working-age populations worldwide (28). Compared with individuals with diabetes without retinopathy, those with DR are more vulnerable to adverse psychological conditions, including depression and anxiety (29). With disease progression, visual function, physical functioning, and social participation continue to decline, thereby negatively affecting overall well-being and health-related quality of life (30). Furthermore, visual impairment associated with DR may restrict work capacity and increase the risk of accidental injuries, imposing substantial economic burdens on both families and society (31). Therefore, understanding the prevalence of DR and its associated factors among individuals with diabetes is crucial for developing targeted prevention strategies, promoting early diagnosis and treatment, and ultimately reducing blindness. This study aimed to evaluate the prevalence of DR and identify its related factors among diabetic individuals in rural Guangxi, China.

The findings indicated a DR prevalence of 22.36% among people living with diabetes residing in rural Guangxi. The research utilized facility-based convenience sampling within counties selected by random methods. This sampling strategy could introduce selection bias by disproportionately representing individuals who actively seek healthcare and are recorded in health management databases. Consequently, these prevalence estimates may overestimate diagnosed, healthcare-seeking diabetic populations. Therefore, caution is advised when extrapolating results to the entire rural diabetic community, particularly undiagnosed or non-attending individuals. The present study also compared its results with prior DR prevalence studies conducted in China. For instance, a rural DR survey in Handan City, Hebei Province, reported a DR prevalence of 43.1% (95% *CI*: 38.0%–48.2%) (32), substantially higher than the findings of this study. The disparity between these two studies may arise from regional differences, such as dietary habits and glycemic management practices in northern and southern China, although these specific data were not obtained in our study. The dietary habits of people in Handan (northern China) are generally spicier, oilier, and saltier than those in Guangxi (southern China). Relevant studies have shown (33) that the overall prevalence of diabetes in Handan is higher. A population-based cross-sectional study carried out by Hu et al. in Liaoning Province, China, demonstrated that the prevalence of DR was 11.9% (95% *CI* = 8.4%–15.4%) (34), lower than the 22.36% reported in this study. This might be because the study in Liaoning employed the 2020 Chinese Diabetes Society Guidelines for Diabetes Diagnosis. These guidelines are stricter within the context of China, which may have resulted in an increased reported prevalence. A study on people living with diabetes in Beijing, northern China, showed that the prevalence of DR was 16.8% (95% *CI* = 14.2%–19.7%) (19), also lower than that found in the present study. Those patients typically have a better understanding of diabetes, superior economic circumstances, and greater access to medical services. Conversely, people living with diabetes in rural Guangxi have relatively restricted access to medical resources, which may significantly contribute to regional differences in the prevalence of DR. Another study on people living with diabetes in rural areas of southern China revealed that the prevalence of vision-threatening DR, diabetic macular edema, and clinically significant macular edema were 2.5%, 2.8%, and 0.9%, respectively, all lower than those reported in this study (20). This further highlights that people living with diabetes in rural Guangxi face significant disadvantages in these aspects, which might explain regional disparities in DR prevalence. This study underscores the disparity in DR prevalence. A meta-analysis of 19 studies conducted in China revealed that the prevalence of DR was higher among rural diabetic patient cohorts compared with urban cohorts (29.1% vs 18.1%) (35). The prevalence of DR among people living with diabetes varies significantly across different geographical regions and populations worldwide. A population-based systematic review (8) reported the prevalence of DR in different regions: 35.90% (95% *CI* = 29.48%–42.87%) in Africa, 33.30% (95% *CI* = 25.29%–42.40%) in North America and the Caribbean, 13.37% (95% *CI* = 6.13%–26.74%) in South and Central America, 16.99% (95% *CI* = 14.13%–20.28%) in Southeast Asia, and 22.27%(95% *CI* = 19.73%–25.03%) globally. The global prevalence reported in that systematic evaluation is similar to the results of this study. These regional differences in the prevalence of DR may be attributed to differences in ethnicity, geographic factors, socio-economic conditions, measurement tools, and data collection methods.

Given these outcomes, targeted interventions are essential to alleviate the DR burden in rural Guangxi. Recommended strategies include: initiating community-based screening programs to facilitate early detection and management, especially targeting elderly and high-risk diabetic individuals; enhancing diabetes education and health promotion activities, emphasizing glycemic and lipid control; expanding affordable diabetes care, including regular monitoring of HbA1c and lipid profiles; and strengthening rural healthcare infrastructures by training local healthcare personnel at township clinics and health centers. The implementation of these interventions could significantly reduce DR prevalence and its complications in rural Guangxi.

Klein et al. demonstrated a significant positive relationship between age and DR (36). However, studies conducted by Cheung et al. (37) and Sasongko et al. (38) identified negative correlations between age and DR occurrence. The likelihood of DR increases with patient age, which is consistent with the findings of Razia (39). This difference may be explained by the fact that Cheung et al. (37) studied healthy individuals, whereas Sasongko et al. (38) focused exclusively on patients with type 1 diabetes aged 12 to 20 years. The Multi-Ethnic Study of Atherosclerosis found that the prevalence of DR did not change significantly with age, possibly due to the inclusion of a large proportion of elderly individuals in the study (40). In contrast, Alramadan et al. found a strong correlation between aging and the risk of DR in elderly populations. The prevalence among individuals aged 60 to 70 years was 38.5%, among those aged 71 to 80 years was 46.8%, and among those aged 81 years and above was 55.4% (41). Therefore, this study further confirms that age is an independent correlate of DR.

This study confirmed that HbA1c is independently associated with DR in adult people living with diabetes in rural areas. HbA1c is a crucial indicator for diabetes management, as it accurately reflects average blood glucose levels over the previous 2–3 months and, to some extent, reflects the progression of DR (42). Elevated HbA1c levels reduce the affinity of erythrocytes for oxygen, making it more difficult for body tissues to effectively utilize oxygen, thereby accelerating the progression of DR (43). Early worsening of diabetic retinopathy (EWDR) further highlights the role of HbA1c in DR risk. A systematic review reported that EWDR occurs in patients with type 1 or type 2 diabetes during intensive glycemic therapy or following pancreas transplantation or bariatric surgery, typically within 3–6 months of rapid glucose improvement (prevalence 10–20%), with a higher likelihood in patients with advanced baseline DR (44). As blood glucose levels increase, leukocytes adhere to the retinal vascular endothelium, leading to endothelial cell death, vascular leakage, and ultimately promoting the formation of microaneurysms and capillary abnormalities. Consequently, insufficient blood and oxygen supply to retinal capillaries occurs, stimulating the expression of angiogenic factors and leading to retinal microvascular disease (45). Recent studies have further highlighted the role of exosome-mediated molecular mechanisms in hyperglycemia-induced retinal microvascular injury. Prolonged exposure to hyperglycemia can modify the molecular cargo of exosomes, including microRNAs, inflammatory mediators, and metabolic regulators, thereby enhancing intercellular communication that drives retinal inflammation, oxidative stress, and endothelial dysfunction. These mechanisms provide a novel biological basis for the association between elevated HbA1c levels and the development of DR (46). To prevent and treat DR, clinicians should encourage people living with diabetes to regularly monitor blood glucose levels, follow a diabetes-appropriate diet, and engage in regular physical activity to achieve glycemic control. Maintaining HbA1c levels below 7% is essential for preventing the progression of DR.

This study identified TG as a factor associated with DR among individuals with diabetes, consistent with the findings reported by Jing et al. (19). This association may be explained by the close relationship between diabetes and dyslipidemia. Abnormal lipid metabolism can impair pancreatic β-cell function, aggravate insulin resistance, enhance lipolysis, and increase circulating free fatty acids and TG levels (47). A study conducted in Taiwan, China, demonstrated that elderly patients with type 2 diabetes and DR had significantly higher TG levels than those without DR (48). Beyond absolute lipid concentrations, increasing evidence indicates that lipid variability also contributes to diabetic microvascular complications (49). Sustained elevation or marked fluctuation in TG levels may induce retinal microvascular injury through oxidative stress, mitochondrial dysfunction, and non-enzymatic glycation. Moreover, the synergistic effects of hyperglycemia and hyperlipidemia can activate inflammatory pathways, promote lipid peroxidation, and enhance apoptosis, thereby accelerating retinal capillary loss and DR progression (50). In contrast, a multi-ethnic cohort study from the United States found no significant association between elevated triglycerides levels and DR among individuals with diabetes (40). Therefore, well-designed randomized controlled trials are needed to further clarify the role of lipid abnormalities in the onset and progression of DR.

## 5 Limitations

This study has several limitations. First, its cross-sectional design among rural patients with diabetes limits causal inference, and the observed associations cannot establish temporality. Additionally, participants were recruited from 15 county-level hospitals, and although unified national diagnostic criteria were applied, inter-hospital differences in laboratory instruments and testing methods for indicators such as HbA1c, uric acid, HDL-C, and LDL-C may have introduced measurement variability. Furthermore, DR was evaluated using routine clinical examinations at each site, and inter-observer or device-related variability cannot be entirely excluded. Because specialized ophthalmic equipment could not be deployed for community-based field screening, only patients who sought hospital-based care were enrolled. Accordingly, caution is warranted when generalizing these findings to the broader rural diabetic population.

Second, the present study employed standard logistic regression, assuming observations were independent, despite the inherent clustering of participants within counties and township health centers. This methodological choice might underestimate standard errors and inflate statistical significance. Thus, future investigations should consider employing multilevel modeling approaches or robust cluster-corrected standard errors.

Third, although numerous demographic, socioeconomic, clinical, and psychosocial variables were included, certain potentially important confounders were not measured or could not be incorporated into analyses. These omitted variables include body mass index, blood pressure and other hypertension-related parameters, physical activity levels, and comprehensive renal function measurements beyond serum creatinine. Consequently, the possibility of residual confounding cannot be entirely excluded. Future research involving larger, more diverse samples is needed to comprehensively evaluate associations between DR and its correlates.

Fourth, the current study did not formally assess logistic regression assumptions, particularly the linear relationship between continuous variables and the log-odds of DR, or the potential multicollinearity among independent variables. Future research could evaluate these assumptions using residual plots, restricted cubic splines, or variance inflation factors, which would strengthen confidence in the model results. Future studies should explicitly address these assumptions to enhance the robustness and validity of regression models.

Finally, evaluating numerous variables through multiple univariate comparisons increases the likelihood of type I errors. Although the current analysis did not formally adjust for multiple comparisons, some significant associations identified may reflect chance occurrences rather than true relationships. Therefore, these findings should be interpreted with caution.

## 6 Conclusion

This study examined diabetes in the rural population of Guangxi. The prevalence of DR among people living with diabetes was 22.36%, with age, elevated triglyceride levels, and HbA1c identified as variables independently associated with DR. These findings highlight the need for targeted interventions, including community-based screening, diabetes education, accessible healthcare services, and strengthened local healthcare infrastructure, to reduce the burden of DR and related complications.

## Data Availability

The original contributions presented in the study are included in the article/supplementary material. Further inquiries can be directed to the corresponding authors.
