# Correction: Factors associated with diabetic retinopathy among patients with diabetes in rural Guangxi, China: a multicenter cross-sectional study

**DOI:** 10.3389/fendo.2026.1802749

**Published:** 2026-02-11

**Authors:** Xiaomin Xian, Jingfeng Chen, Ziqiang Li, Qiuping Zheng, Xiaoxue Lei, Chaoqun Bai, Yanping Zhang, Guifen Fu

**Affiliations:** 1 Nursing Department, Faculty of Chinese Medicine Science, Guangxi University of Chinese Medicine, Nanning, China; 2 Department of Geriatric Endocrinology and Metabolism, Guangxi Academy of Medical Sciences and the People’s Hospital of Guangxi Zhuang Autonomous Region, Nanning, China; 3 Department of Nursing, Guangxi Academy of Medical Sciences and the People’s Hospital of Guangxi Zhuang Autonomous Region, Nanning, China

**Keywords:** correlates of DR, diabetes, prevalence, retinopathy, rural

Affiliation “^2^Department of Geriatric Endocrinology and Metabolism, Guangxi Academy of Medical Sciences and the People’s Hospital of Guangxi Zhuang Autonomous Region” was omitted for authors Jingfeng Chen, Ziqiang Li, Qiuping Zheng, Xiaoxue Lei, Chaoqun Bai and Yanping Zhang. This affiliation has now been added for these authors.

Authors “Jingfeng Chen, Ziqiang Li, Qiuping Zheng, Xiaoxue Lei, Chaoqun Bai and Yanping Zhang” were erroneously assigned to affiliation “^1^Nursing Department, Faculty of Chinese Medicine Science, Guangxi University of Chinese Medicine”. This affiliation has now been removed for these authors.

The original version of this article has been updated.

